# Supplementary material for: Inflammatory factors and risk of lung adenocarcinoma: a Mendelian randomization study mediated by blood metabolites
Source: Front Endocrinol (Lausanne). 2024 Aug 27;15:1446863. doi: 10.3389/fendo.2024.1446863 (PMC11384989; doi:10.3389/fendo.2024.1446863)
Supplement: Supplementary file 1 [file DataSheet1.zip › supplementary files/S2/GCST90200978/sensitivity-analysis.pdf]

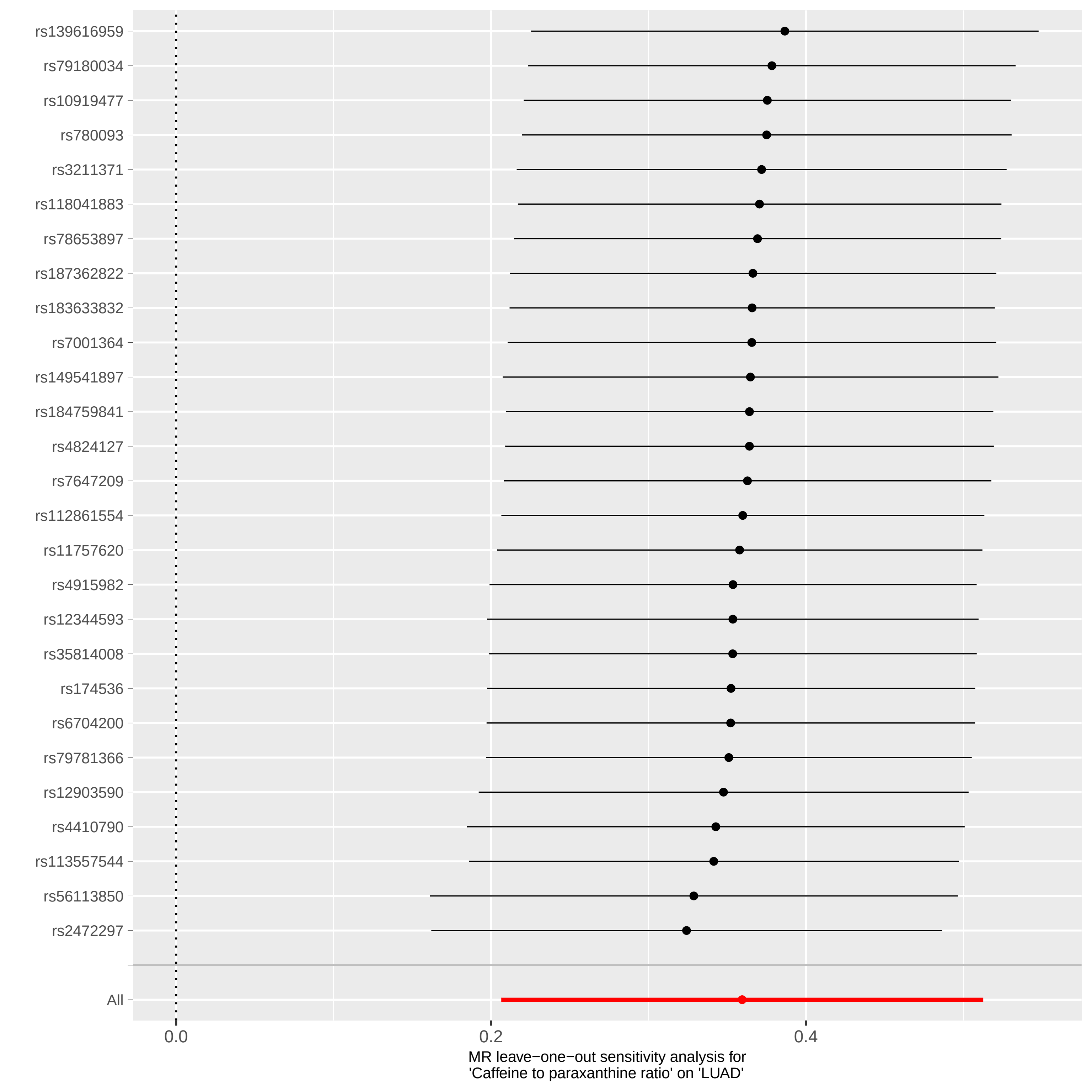
rs139616959
rs79180034
rs10919477
rs780093
rs3211371
rs118041883
rs78653897
rs187362822
rs183633832
rs7001364
rs149541897
rs184759841
rs4824127
rs7647209
rs112861554
rs11757620
rs4915982
rs12344593
rs35814008
rs174536
rs6704200
rs79781366
rs12903590
rs4410790
rs113557544
rs56113850
rs2472297
All
0.0
0.2
0.4
MR leave−one−out sensitivity analysis for
'Caffeine to paraxanthine ratio' on 'LUAD'
